# A Comparative Study of the ProSeal Laryngeal Mask Airway Versus Endotracheal Tube in Neonates With Anorectal Malformations

**DOI:** 10.7759/cureus.16798

**Published:** 2021-07-31

**Authors:** Durga P Kannojiya, Shefali Gautam, Vinod K Srivastava, Gyan Prakash Singh, Ram g Maurya, Anita Malik, Jyotsna Agarwal, Monica Kohli

**Affiliations:** 1 Department of Anaesthesiology and Critical Care, King George's Medical University, Lucknow, IND

**Keywords:** proseal lma, endotracheal tube, arm, anaesthesia, airway

## Abstract

Background: Laryngeal mask airways (LMAs) are widely used in paediatric anaesthesia. However, LMA use in neonatal age groups (younger than seven days) is limited because many anaesthesiologists prefer to use endotracheal tube in neonates. In this study, we compared the ProSeal LMA and endotracheal tube by measuring their performance, including ease of insertion via number of attempts for placement of device, total effective time for intubation and extubation, hemodynamic responses and perioperative complications.

Methods: In this prospective randomized study, 70 patients (neonates) weighing >2.5 kg, with American Society of Anaesthesiologists (ASA) classification grade 4 requiring emergency surgery for anorectal malformation were enrolled and divided into two groups. After induction, patients' airways were secured with either ProSeal LMA size 1 (Group I) or endotracheal tube (Group II). Anaesthesia was maintained on oxygen and sevoflurane with muscle relaxant atracurium.

Results: Demographic and surgical data were similar between the two groups. The ProSeal LMA insertion time was shorter than endotracheal intubation. Hemodynamic variations were less in the ProSeal LMA group as compared to the endotracheal tube group. The total time for removal of airway devices from the end of surgery for the ProSeal group was lower than that for the endotracheal intubation group. Postoperative complications were less in the ProSeal group as compared to the endotracheal group.

Conclusions: The ProSeal LMA can be a better alternative to the endotracheal tube in neonates due to the ease of insertion, lesser changes in hemodynamic parameters and minimal postoperative complications.

## Introduction

Children have been the earliest patrons of anaesthesiology from its earliest clinical applications of surgical anaesthesia [1]. Airway management in children becomes more challenging because of the differences in airway anatomy. In comparison to older children, adolescents and adults, anatomy of neonatal upper airway structures is different and endotracheal intubation becomes a challenge.

To avoid such adverse complications associated with endotracheal intubation, the first supraglottic airway device, the laryngeal mask airway (LMA), was designed in 1981 by Dr. Archie Brain [2].

The latest addition to the family of supraglottic airway devices in the paediatric age group was the second-generation devices. In 2000, the ProSeal LMA, a second-generation supraglottic airway device was introduced. It is the modified form of the classic LMA. It consists of a wire-reinforced airway tube and a parallel drainage tube. The gastric drainage tube is placed next to the main airway tube and forms a channel for regurgitated gastric contents, thus preventing gastric insufflation and pulmonary aspiration [2,3].

The ProSeal LMA is frequently used in the paediatric age group as an alternative to the endotracheal tube, but there are limited studies in neonates (younger than seven days) for surgical procedures.

Therefore, we chose to conduct a comparative study between the ProSeal LMA 1 and endotracheal tube in neonates with anorectal malformation with respect to the ease of insertion via number of attempts for the placement of the device, total effective time for intubation and extubation, hemodynamic responses and perioperative complications.

## Materials and methods

After the approval of the institutional ethics committee, this prospective randomized study was conducted from August 2018 to July 2019 in the Department of Anaesthesiology, King George’s Medical University, Lucknow, India. A total of 70 neonates (younger than seven days), weighing >2.5 kg, with American Society of Anaesthesiologists (ASA) classification grade 4 with high anorectal malformations requiring emergency surgery under general anaesthesia were enrolled. Those with the guardian not giving consent, with any other congenital anomaly, preterm, low birth weight, i.e., <2.5 kg, and patients having systemic illnesses like respiratory dysfunction, hemolytic disorder, or cardiac pathology were excluded from our study. All patients were randomly divided into two groups of 35 each using computer-generated random tables.

In Group I, patients’ airway was secured with ProSeal LMA 1 and in Group II patients’ airway was secured with an endotracheal tube. Patients were kept on fasting for four and six hours for breast milk and formula milk, respectively, as these were semi-emergency cases and nasogastric tube was already placed for decompression of stomach. After taking the patient to the operation room, nasogastric tube aspiration was done and intravenous line secured. Monitors like precordial stethoscope, pulse oximeter, electrocardiogram, noninvasive blood pressure monitor and temperature probe were attached and baseline vitals recorded. Premedication was done with 0.2 mg atropine, and after pre-oxygenation with 100% oxygen for five minutes using the modified Jackson-Rees circuit of Ayre’s T-piece, fentanyl 2 mcg/kg body weight was given to both groups. Induction was done with thiopentone 3 mg/kg body weight and succinylcholine 1 mg/kg body weight. After rapid sequence induction (without ventilation and without cricoid application), patient airway was secured by same consultant anesthetist with either ProSeal LMA 1 or endotracheal tube.

ProSeal LMA size 1 without an introducer was selected for Group I patients; the cuff was fully deflated and the posterior surface of the ProSeal LMA was well lubricated with 2% lignocaine jelly. Patient’s head was maintained in the sniffing position for placement of the LMA and the cuff was inflated with ≤4 ml of air as recommended by the manufacturer (Teleflex Inc., Morrisville, NC). After obtaining an effective airway (defined as normal thoracoabdominal movements, bilaterally equal audible breath sounds on auscultation and a regular waveform on capnograph), the ProSeal LMA 1 was fixed by taping it over the chin. Gastric tube number 8 was inserted through a drain tube. In Group II patients, endotracheal intubation was done using a 2.5/3.0 mm internal diameter uncuffed PVC tube, and gastric tube number 8 was inserted through nasal route and each group of patients was put on controlled mode of ventilation . Patients were maintained on oxygen and sevoflurane with a 0.5 mg/kg loading dose of atracurium muscle relaxant and intraoperative paracetamol 15 mg/kg body weight given before the completion of surgery. The analgesic dose of a caudal epidural block was given to all patients for post-operative pain relief at the end of surgery; anaesthetic agents were discontinued and neuromuscular blockade was reversed with neostigmine and atropine

The following parameters were assessed: (a) effective airway time from the introduction of the airway device up to confirmation by end-tidal carbon dioxide (EtCO_2_); (b) hemodynamic parameters (heart rate, systolic blood pressure [SBP], diastolic blood pressure [DBP], mean arterial pressure [MAP]), SpO_2_, EtCO_2_ from the time of induction to the end of surgery and up to 10 min after the removal of airway devices in both groups​​​​​; (c) total time of extubation from the end of surgery to the removal of the airway device and (d) airway complications like bronchospasm, coughing, laryngospasm, desaturation and breath holding.

Statistical analysis

Sample size was calculated on the basis of the proportion of complications in the two study groups and using the formula of sample size calculation. The results were analysed using descriptive statistics and making comparisons among various groups. Discrete (categorical) data were summarized as proportions and percentages and quantitative data were summarized as means ± SDs. The data were analysed using SPSS Statistics, version 20 (IBM Corp, Armonk, NY).

## Results

Patients in both groups were comparable in view of age, weight, gender and duration of surgery. The mean duration of surgery was 73.86 ± 9.93 min in Group I and 75.09 ± 15.09 min in Group II.

There was no significant difference regarding the baseline pulse rate, saturation and respiratory rate before the induction of anaesthesia. The airway was secured in 98% of patients in the first attempt and in only 2% in the second attempt in the ProSeal LMA group, while in the endotracheal intubation group, 88% of patients had the airway secured in the first attempt and 12% in the second attempt (Table 1).

**Table 1:** Number of attempts at insertion PLMA, ProSeal laryngeal mask airway; ET, endotracheal

Attempts	Airway secured, Group I (PMLA), %	Airway secured, Group II (ET tube), %
1st	98.00	88.00
2nd	99.00	96.00

The time taken to secure the airway, from the introduction of the airway device to the confirmation by EtCO_2_ was significantly lower in Group I (10.20 ± 2.59 sec) than Group II (49.57 ± 11.90 sec), p<0.001, and this might be due to difficult airway anatomy in the paediatric age group. The total time for extubation from the end of surgery to the removal of airway devices was more in Group II than Group I (p<0.001) (Table 2).

**Table 2:** Comparison of the total time of intubation and extubation between the groups

Variable	Group I		Group II		t-value	p-value
	Mean	SD	Mean	SD		
Total time for securing airway devices from induction (sec)	10.20	2.59	49.57	11.90	-19.12	<0.001
Total time for the removal of airway devices from the end of surgery (sec)	253.71	48.45	721.71	175.52	-15.21	<0.001

Both groups were comparable in hemodynamic parameters before securing airways in terms of heart rate, systolic blood pressure, diastolic blood pressure, mean blood pressure and saturation.

**Figure 1:**
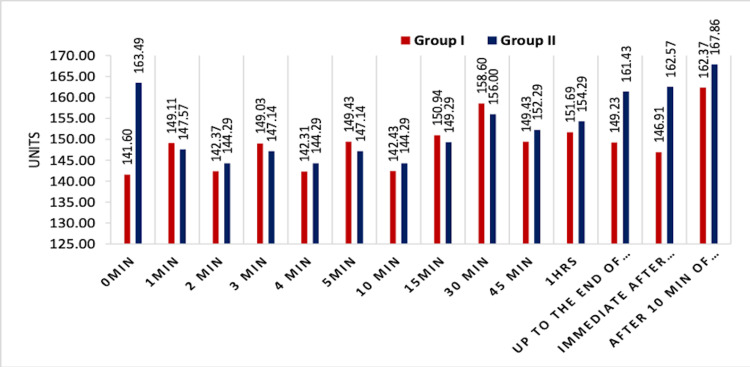
Comparison of the heart rate between the two groups

However, a significant difference was seen in the heart rate at 0 min (immediately after securing airway devices) in Group I (141.60 ± 14.61 bpm) and Group II (163.49 ± 12.65 bpm) that was highly significant (p≤0.001). Intraoperatively, the heart rate was comparable in both of groups. At the end of surgery in Group I, the heart rate was 149.23 ± 29.67 bpm and in Group II, it was 161.43 ± 11.54 bpm, that was significant (p=0.027). Immediately after extubation, in Group I, the heart rate was 146.91 ± 11.05 bpm, and in Group II, 162.57 ± 20.31 bpm, which was highly significant (p=<0.001). Hence, a significant difference was seen in the heart rate immediately after securing the airway device and removal of airway device (Figure 1).

**Figure 2:**
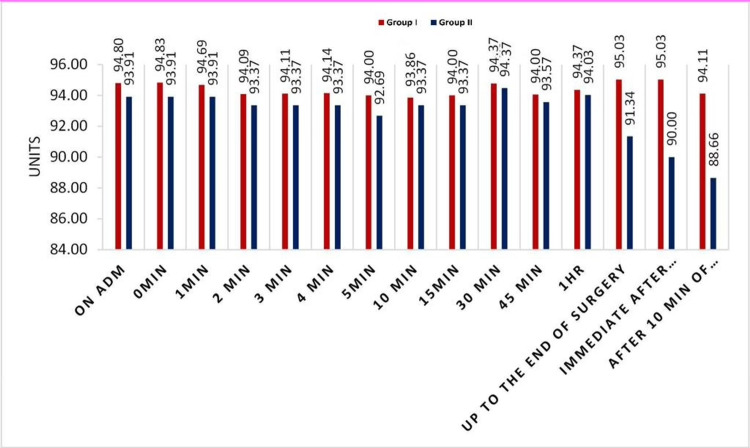
Comparison of SpO2 between the two groups

There was no significant difference in the mean SpO_2_ (%) and EtCO_2_ levels recorded at different time points between the two groups, except that after the removal of the airway device, eight patients desaturated in the endotracheal tube group and one patient in the ProSeal group (SpO_2_ 90%). But none of the patients were reintubated in our study but managed successfully by providing oxygen by mask only (Figure 2).

**Figure 3:**
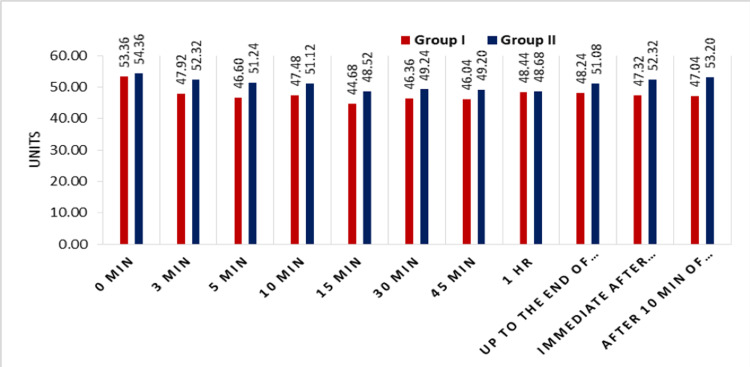
Comparison of mean arterial pressure between the two groups

In our study statistically significant variations were found in SBP, DBP and MAP at different time points, but were not clinically significant (Figure 3).

Regarding postoperative complications, bronchospasm was seen in six patients in Group II and none in Group I (p=0.010), coughing was seen in three patients in Group II and none in Group I, laryngospasm was seen in one patient in Group II and none in Group I, desaturation developed in eight patients in Group II in which six patients immediately desaturated after extubation and two after five minutes of extubation, while breath holding developed in one patient in Group I and two patients in Group II. One patient desaturated up to 90% in Group I after five minutes of the removal of the Proseal LMA (p=0.012). None of the patients was reintubated (Table 3).

**Table 3:** Distribution of cases according to complications

Complication	Group I		Group II		Chi-square	p-value
	No.	%	No.	%		
Breath holding	1	2.9%	2	5.7%	0.36	0.552
Bronchospasm	0	0.0%	6	17.1%	6.56	0.010
Coughing	0	0.0%	3	8.6%	3.13	0.077
Laryngospasm	0	0.0%	1	2.9%	1.01	0.314
Desaturation	1	2.9%	8	22.9%	6.25	0.012
Total	2	5.8%	20	57.14%		

## Discussion

Numerous supraglottic airway devices have been introduced in the past few years, in the quest to provide better alternatives to intubation of the trachea. But there are limited studies done in neonatal patients using the ProSeal LMA. The modifications were designed to enable the separation of the gastrointestinal tract and respiratory tract, improving the airway seal, enable controlled ventilation and diagnose misplacement.

Ozden et al. conducted a study in the paediatric age group patients between 1 to 24 months of age to compare airway device insertion and post-operative complications [4]. They observed that the time required to secure the airway as well as the number of attempts made to insert the airway device in the ProSeal group were less as compared to the endotracheal tube group, and the results were consistent with present study where the insertion time of the ProSeal LMA was less as compared to endotracheal intubation.

In another study by Lalwani et al. conducted in the age group of two- to eight-year-old patients, it was found that the success rate for the placement of the ProSeal LMA and endotracheal tube in the first attempt was 83.33% and 96.67%, respectively [5]. The success rate in the second attempt in the ProSeal LMA and endotracheal groups was 16.6% and 3.33%, respectively, which showed that the time required for insertion was more in the Proseal LMA group as compared to endotracheal intubation. Thus, this study did not support our present study where the ProSeal LMA was inserted more successfully in the first attempt as compared to endotracheal intubation. In our study, the age of patients was less than seven days and body weight was more than 2.5 kg, but in the above-mentioned study, both age and weight were more and they used ProSeal LMA with size above 1. Hence, in the present study, the effective airway time was less in the ProSeal LMA group as compared to the endotracheal tube group. It could be due to the age less than seven days (early neonates), smallest size ProSeal LMA (size 1) that lacks dorsal cuff making insertion easier and individual expertise when compared to endotracheal intubation.

Both groups were comparable in hemodynamic parameters before securing the airway in terms of heart rate, SBP, DBP, MBP and saturation. However, a significant difference was seen in the heart rate at 0 min (immediately after securing airway devices) in Group I (141.60 ± 14.61 bpm) and Group II (163.49 ± 12.65 bpm) that was highly significant (p≤0.001). Regarding the SBP (in mm of Hg), we found that in Group I (ProSeal LMA), a significant decrease was observed as compared to Group II (endotracheal tube ) at all time points. It might be due to the fact that sympathetic stimulation is more for the endotracheal tube as compared to the ProSeal LMA. Statistically significant variations were found in SBP, DBP and MAP at different time points, but it was clinically not significant.

Our results were consistent with the previous study done by Lalwani et al., wherein the increase in the pulse rate was statistically significant in the endotracheal tube group at just after insertion and even after 10 min of insertion, and they also found that there was a statistically significant (p<0.05) decrease in the mean SBP from the baseline value at five minutes after the placement of the ProSeal LMA (Group A). The mean SBP also decreased from the baseline mean SBP at five minutes after endotracheal intubation (Group B) (p>0.05), but in the ProSeal group, it was significantly decreased. They found that blood pressure was significantly reduced in the Proseal LMA group as compared to the endotracheal tube group. The increase in the mean DBP and MAP was statistically significant (p<0.05) in Group II compared to Group I, but clinically it was not significant. The results were consistent with our study.

In another prospective randomized comparative study done by Patel et al. to see the use of ProSeal LMA and endotracheal tube for airway management in children under general anaesthesia, it was found that the hemodynamic response to the ProSeal LMA was less as compared to laryngoscopy and intubation, because there was a significant rise in the heart rate and blood pressure in the endotracheal tube group as compared to the ProSeal LMA group [6]. The postoperative complications comprised mild sore throat, coughing, vomiting and hypoxemia seen in children who were intubated. The results were consistent with our study though the age group was higher.

As the ProSeal LMA is a less invasive airway device, postoperative airway-related complications were less with the ProSeal LMA than with endotracheal tube, because while using a classic LMA (cLMA), the cuff pressure decreases mucosal perfusion, thus increasing the incidence of post-operative airway morbidity. Although tracheal mucosal cuff pressure is not clearly defined for paediatric patients for endotracheal tube, ProSeal LMA cuff pressure was considered to contribute to the decreased incidence of post-operative complications. A lower incidence of post-operative complications was also an advantage of the ProSeal LMA over the endotracheal tube. There was no incidence of aspiration in either group during the induction of anaesthesia, intraoperative period or after the removal of the respective airway device [7,8].

Overall, paediatric anaesthesia has remained a challenge for anaesthesiologists since the beginning of surgery. Based on our study, the ProSeal LMA seems to be a good alternative to endotracheal intubation in neonates. Several design and performance features seem to provide protection against aspiration. The paediatric ProSeal LMA lacks the additional dorsal cuff that makes its insertion easier. When positioned correctly, the tip of the device forms a high pressure seal with the oesophageal inlet. In addition to this, the ProSeal LMA doesn’t necessarily require the use of muscle relaxants. Hemodynamic parameters were more stable with the use of the ProSeal LMA as compared to the endotracheal tube, both during the insertion and removal of the airway device. The reason could be that patients who were intubated (Group II) needed a deeper plane of anaesthesia, thus requiring more time to recover from the effect of general anaesthesia. On the contrary, those patients whose airway was secured by the ProSeal LMA (Group I) were maintained in a relatively less deeper plane of anaesthesia to blunt airway reflexes and therefore, required less time for the removal of the ProSeal LMA.

The incidence of postoperative complications with the ProSeal LMA is also less when compared to endotracheal intubation [9]. In neonates with anorectal malformation, there is an increased risk of aspiration. However, the previous concept of rapid sequence induction (RSI) remains controversial. Because of potential airway obstruction and the associated technical problems (the adult hand restricts mouth opening and interferes with proper positioning of the handle of the laryngoscope), the reliability and feasibility of cricoid pressure have been questioned by paediatric anaesthesiologists [10-14]. Moreover, the choice of the drug, ventilation during apnoeic episode, patient position and application of cricoid pressure in RSI remain controversial. Because the application of cricoid pressure hampers the insertion of the ProSeal LMA, its use solves the problems associated with RSI to a certain extent. The large bulk of the ProSeal LMA occupies the pharynx and perilaryngeal tissues decreasing the risk of aspiration, and the drain tube, which runs through the device from the tip to the proximal end, vents any gas leaking into the oesophagus, reducing the risk of gastric inflation.

Despite all the effective measures taken, we came across few limitations in the present study. Although an adequate sample size was taken, the power of the study could have increased if it was performed on a larger sample size. Many of the secondary outcomes were subjective and hugely depended on the analyser. Also, it was a single-centre study.

## Conclusions

Keeping the entire study into consideration, we can conclude that the ease of intubation and insertion time of the airway device was more convenient and less time consuming in the ProSeal LMA group as compared to the endotracheal tube group. Hemodynamic variations and the postoperative complications comprising mild sore throat, coughing and hypoxemia were less in the ProSeal LMA group as compared to the endotracheal group. Therefore, the ProSeal LMA should be considered as a good alternative to the endotracheal tube in neonates with anorectal malformation as the use of ProSeal LMA is limited in neonates for surgery.

However, we suggest further multi-centred studies with larger sample sizes to conclude more firmly on better and safer means for securing the airway in the neonatal age group.
